# Critical Appraisal of “Management of hydrocephalus associated with posterior fossa tumors in adults: results of a prospective study”

**DOI:** 10.1097/MS9.0000000000004139

**Published:** 2025-10-16

**Authors:** Asra Amjad, Syeda Tayyaba Batool, Umair Ali, Muddassir Khalid

**Affiliations:** a Islamic International Medical College, Riphah International University, Rawalpindi, Pakistan; b Department of Medicine, Azad Jammu and Kashmir Medical College, Muzaffrabad, Azad Jammu and Kashmir, Pakistan; c Department of Pharmacy, University of Swabi, Swabi, Pakistan; d Department of Medicine, Nishtar Medical University, Multan, Pakistan

*To the Editor,*

The recent paper on predictors of pre-resection hydrocephalus in posterior fossa tumors, published in *Neurosurgical Review*, caught our attention. Although the study addresses a very pertinent clinical issue, some methodological and interpretive elements warrant closer examination. Because the cerebrospinal fluid (CSF) routes are blocked, hydrocephalus is often a complicating factor for tumors of the posterior cerebral fossa (PCF). Due to anatomical vulnerability in the pediatric population, the prevalence is significantly higher in children (up to 100%) than in adults (30–40%)^[1,2]^. This notable disparity highlights the need for age-specific risk classification, since children continue to be disproportionately at risk. Hemangioblastomas, ependymomas, medulloblastomas, and large vestibular schwannomas are among the tumors closely linked to hydrocephalus^[3]^. Risk assessment can be improved by focusing on these tumor subtypes, although tumor biology and presentation often present additional complications. Clinical and radiological indicators of pre-resection hydrocephalus were recently found in a multicenter cohort of 421 patients. Symptoms of elevated intracranial pressure, ataxia, cognitive impairment, tumor volume, and peritumoral vasogenic edema were all significant factors. Schwannomas were inversely linked with risk, whereas malignant histology significantly raised it.

It is interesting to note that isolated cranial nerve dysfunction, such as facial numbness or hearing loss, appears protective, likely because it is often discovered sooner^[4]^. These results suggest that the clinical appearance may have predictive significance in its own right and is not merely a consequence of tumor size. A predictive scoring model that combined tumor volume with four clinical variables: ataxia, elevated intracranial pressure, peritumoral edema, and cognitive impairment, achieved 81.2% specificity and 87.5% sensitivity at a threshold score of ≥4. These instruments could aid in the early diversion of CSF fluid before tumor excision and the stratification of high-risk patients^[4]^. External validation among diverse groups remains crucial, as relying solely on one cohort for validation raises questions about generalizability. In addition to direct ventricular blockage, hydrocephalus in these patients is known to be caused by venous sinus compression and altered intracranial hemodynamics^[5]^. This more comprehensive understanding of pathophysiology emphasizes the necessity of multimodal evaluation, pointing out that a single cause rarely explains hydrocephalus. By identifying these pathways and using proven clinical predictors, perioperative care for PCF tumors can be improved. However, prospective validation, integration with surgical decision-making, and alignment with treatment pathways for adults and children will be necessary for translation into ordinary practice. Lastly, hydrocephalus is typically associated with PCF tumors, especially those that occur in children. Key factors that can be used to identify high-risk patients include tumor volume, ataxia, and cognitive impairment. Using both direct and indirect causes of hydrocephalus, the predictive scoring model can guide early treatments to improve perioperative care and outcomes. In summary, even if the prediction model put forth makes significant strides, further research is needed to confirm and enhance it across diverse populations, ensuring its suitability and dependability for guiding neurosurgical decision-making.

This Letter to the Editor complies with the TITAN guideline^[6]^.

## Data Availability

Not applicable.

## References

[1] Schmid UD Seiler RW. Management of hydrocephalus associated with posterior fossa tumors in adults: results of a prospective study. Acta Neurochir (Wien) 1986;82:115–18.3788673

[2] Kumar R Bhowmick U Kalra SK. Hydrocephalus and medulloblastoma: management strategies and long-term follow-up. Childs Nerv Syst 1996;12:588–93.

[3] Culley DJ Berger MS Shaw D. Hydrocephalus complicating posterior fossa tumors in children: a review of 117 cases. Neurosurgery 1994;34:402–08. 8190214 10.1227/00006123-199403000-00003

[4] Kanjanakangwankul P Sitthinamsuwan B Ngamsombat C. Predictors of pre-resection hydrocephalus in posterior cranial fossa tumors: development of a predictive scoring model. Neurosurg Rev 2025;48:607. 40825885 10.1007/s10143-025-03752-2 PMC12361321

[5] Bateman GA Szczudlik A Klimkowicz A. The role of venous sinus compression in the pathophysiology of hydrocephalus. Clin Neurol Neurosurg 2003;105:87–95.12691796

[6] Science P London UK Agha R. Transparency In The reporting of Artificial INtelligence – the TITAN guideline. Premier J Sci 2025;10:100082.

